# Which Form of Medical Training is the Best in Improving Interns’ knowledge Related to Advanced Cardiac Life Support Drugs Pharmacology? An Educational Analytical Intervention Study Between Electronic Learning and Lecture-Based Education

**DOI:** 10.5812/aapm.15546

**Published:** 2014-02-08

**Authors:** Manouchehr Khoshbaten, Hassan Soleimanpour, Alireza Ala, Samad Shams Vahdati, Kimia Ebrahimian, Saeid Safari, Samad EJ Golzari, Fariba Salek Ranjbarzadeh, Robab Mehdizadeh Esfanjani

**Affiliations:** 1 Medical Education Research Center, Tabriz University of Medical Sciences, Iran; 2 Cardiovascular Research Center, Tabriz University of Medical Sciences, Tabriz, Iran; 3 Department of Emergency Medicine, Tabriz University of Medical Sciences, Tabriz, Iran; 4 Students’ Research Committee, Tabriz University of Medical Sciences, Tabriz, Iran; 5 Department of Anesthesiology and Critical Care Department, Iran University of Medical Sciences, Tehran, Iran; 6 Medical Philosophy and History Research Center, Tabriz University of Medical Sciences, Tabriz, Iran; 7 Neurosciences Research Center, Tabriz University of Medical Sciences, Tabriz, Iran

**Keywords:** Cardiopulmonary Resuscitation, Emergency Medicine, Education

## Abstract

**Background:**

Conventional educational systems seem to be improper throughout the cardiopulmonary resuscitation (CPR) teaching process. The most common causes of failed resuscitation are unfamiliarity with cardiopulmonary resuscitation algorithms, poor performance of leader of the CPR team and lack of skilled personnel, coordination among members during resuscitation, and responsibility of staff.

**Objectives:**

Electronic learning, as a new educational method is controversial issue in medical education for improving physicians’ practical knowledge and it is inevitable that further research on its effectiveness should be done.

**Materials and Methods:**

The present study is a prospective, pre- and post-educational, cross-sectional research, in which 84 interns were randomly divided into two groups. pre- and post- educational interventions that took place in the Department of Emergency Medicine, interns were evaluated by 21 multiple choice questions related to American Heart Association guidelineson cardiopulmonary resuscitation drugs. Questions were assessed in terms of routes for CPR drugs administration, CPR drug dosage forms, clinical judgment and appropriate CPR drug administration, and the alternative drugs in emergency situations. Data were analyzed by generalized estimating equations regression models and P < 0.05 was considered statistically significant.

**Results:**

Evaluating the effectiveness of both educational methods revealed that the mean answering score for 21 questions before education was 7.5 ± 2.6 and no significant difference was observed in groups (P = 0.55). However, after education, the average scores significantly increased to 11.0 ± 3.9 (P < 0.001). Electronic learning method was not associated with considerable increase in the knowledge of interns in this group compared with the lecture-based group (P = 0.49).

**Conclusions:**

No significant differences were observed between electronic learning and lecture-based education in improving interns’ knowledge of CPR drugs.

## 1. Background

As a life-saving intervention, CPR has always been of great importance in medical training. However, the process of learning, durability, and remembering the stages seem to be difficult (1-3). According to the statistical report of the United States Department of Health and Medical Education, about three hundred thousand people annually die due to cardiovascular diseases. Surprisingly, it is believed that 20 to 30 percent of these lives could have been saved with the administration of proper CPR (4-7). Although there are no reliable statistics on successful resuscitation rates in Iran, a previous study showed that only 9.7 percent of medical staff possessed the required skills (8, 9). The most common causes of failed resuscitation are unfamiliarity with cardiopulmonary resuscitation algorithms, poor performance of leader of the CPR team and lack of skilled personnel, coordination among members during resuscitation, and responsibility of the staff (10-14). These facts are indicative of defects in the process of learning cardiopulmonary resuscitation. Thus, paying attention to modern educational methods and applying them may result in the improvement of the process of learning and stabilization of what has already been learnt.

Throughout resuscitation process, the main challenge in patients’ survival is that the surrounding personnel, nurses, and even primary health care physicians suffer from lack of required and appropriate knowledge of interventions. Consequently, these groups, especially professionally involved with medical care and treatment, must pass the training courses of resuscitation and pre-hospital and in-hospital care to increase their skills (15-24).

## 2. Objectives

Since the electronic learning is one of the modern methods of education and it is being implemented in some countries, the present research was carried out to study the effectiveness of this educational method in improving interns’ ( introduced to the Department of Emergency Medicine for passing a one-month course) knowledge about advanced pharmacology of drugs in cardiopulmonary resuscitation.

## 3. Materials and Methods

This study was a prospective pre- and post-educational, cross-sectional research, conducted to examine the performance of interns, introduced to the Department of Emergency Medicine by Deputy of General Practice (GP) Program, to pass a one-month didactic Emergency Medicine Course. The inclusion criterion was the introduction of interns to the Department of Emergency Medicine and the exclusion criteria were unwillingness to engage in this study and not to take part in the pre or post-educational exams. As no similar studies were found in the literature review, the approximate sample size of 84 interns was considered respecting the average number of 10.5 ± 1.41 interns introduced to our Emergency Department monthly. Accordingly, after confirmation of Ethics Committee of the Tabriz University of Medical Sciences, Tabriz, Iran and obtaining written informed consents, 84 interns were included in this research within 8 months from April 2012 to March 2013. These interns had taken no previous educational courses related to cardiopulmonary resuscitation. The introduced interns were allocated into two groups using simple randomization based on their introduction to the Emergency Department. The names of 8 months were written on balls, later pulled out from a bag that inside could not be seen:

Group I included 41 interns who underwent electronic learning and 43 interns were included in group II and underwent lecture-based education. For both groups, the process was initiated by a pre-test in parallel, based on American Heart Association (AHA) multiple choice questions (21 questions as are shown in Box 1) (25). We used a translated questionnaire in the study. In order to evaluate the validity, the questionnaires were distributed among 10 professors of the Emergency Medicine and Cardiology Departments. Their comments were collected and the validity of the questionnaire was confirmed. In order to evaluate the reliability of the questionnaire, a pre-test on 40 students was conducted and alpha of 0.92 was calculated for the study using Cronbach’s alpha, indicative of an acceptable reliability. The test was conducted upon the basis of the following areas:

1. Routes for drug administration (questions 1, 7, 12, 17)
2. CPR drug dosage forms (questions 1, 9, 10, 11, 14, 15, 18)
3. Clinical judgment and appropriate drug administration (questions 3, 4, 11, 12, 13, 14, 19, 20, 21)
4. Alternative drugs in emergency situations (question 8)

Then, an electronic software, based on introduction of 45 CPR drugs, confirmed by AHA, was distributed among interns of group I. In this software, for each drug, indications, contraindications, drug dosage, and precautionary principles were explained in a separate electronic format. Additionally, an eleven-minute educational clip was prepared based on AHA guidelines on advanced cardiovascular life support (ACLS) and usage of drugs was included in the curriculum of this software. Also, the manual of the educational software was provided. A six-hour lecture-based educational course relating to use of 45 CPR drugs approved by AHA, was also held for interns of group II within 3 consecutive weeks (2 hours per week). At the end of the courses (after one month), both groups participated in the post-educational exam with the same questions as pre-educational exam. Both pre and post-educational exams were performed for both groups using the pen and paper approach.

The data were analyzed using descriptive and deductive statistical approaches by SPSS version 17.01 (SPSS Inc., Chicago, Illinois) software. Answers were scored as binary; this means that if someone could answer each question correctly, he/she would receive a positive score; otherwise, no score would be given. According to the study, designed based on before and after manner, the estimations were calculated by generalized estimating equations regression. Time (pre and post) and group (electronic learning and lecture-based education) variables were included in the model. In order to compare the mean scores of the two groups before the intervention, we used independent samples t-test and the obtained t-test was reported. Finally, P < 0.05 was considered as statistically significant.

**Box 1.** Advanced Cardiovascular Life Support Drug Questionnaire

**1. Which of the following is the most accurate statement regarding the administration of vasopressin during cardiac arrest?**
a. Vasopressin is indicated for VF and pulseless VT prior to the delivery of the first shock
b. The correct dose of Vasopressin is 40 U administered IV ^a^ or IO ^a^
c. Vasopressin is recommended instead of epinephrine for the treatment of asystole
d. Vasopressin can be administered twice during cardiac arrest
**2. Your patient has been intubated. IV/IO access is not available. Which combination of drugs can be administered by the endocrinal route of administration?**
a. Amiodarone, lidocaine, epinephrine
b. Epinephrine, vasopressin, amiodarone
c. Lidocaine, epinephrine, vasopressin
d. Vasopressin, amiodarone, lidocaine
**3. Which of the following statements about the use of magnesium in cardiac arrest is the most accurate?**
a. Magnesium is indicated for VF/pulseless VT associated with torsades de pointes
b. Magnesium is indicated for shock-refractory monomorphic VT
c. Magnesium is contraindicated in VT associated with a normal QT interval
d. Magnesium is indicated for VF refractory to shock and amiodarone or lidocaine.
**4. A patient with a possible acute coronary syndrome has ongoing chest discomfort unresponsive to 3 sublingual nitroglycerine tablets. There are no contraindications and 4 mg of morphine sulfate was administered. Shortly, BP ^a^ falls to 88/60 and the patient complains of increased chest discomfort. You would:**
a. Give an additional 2 mg of morphine sulfate
b. Start dopamine at 2 ụg/kg per minute and titrate to BP 100 systolic.
c. Give nitroglycerin 0.4 mg sublingually
d. Give normal saline 250 mL to 500 mL fluid bolus
**5. A patient has a rapid irregular wide-complex tachycardia. The ventricular rate is 138. He is asymptomatic with a BP of 110/70. He has a history of angina. Which of the following actions is recommended?**
a. Give lidocaine 1 - 1.5 mg IV bolus
b. Immediate synchronized cardioversion
c. Seek expert consultation
d. Give adenosine 6 mg IV bolus
**6. A 62-yearold man suddenly began to experience difficulty speaking and left-sided weakness. He is brought to the ER ^a^. He meets initial criteria for fibrinolytic therapy and a CT scan of the brain is ordered. Guidelines for antiplatelet and antithrombotic therapy are:**
a. Administer heparin if CT scan is negative for hemorrhage
b. Give aspirin 160 mg and clopidogrel 75 mg orally
c. Administer aspirin 160-325 mg orally chewed, immediately
d. Do not give aspirin for at least 24 hours if tPA is administered
**7. A patient is in cardiac arrest. VFib ^a^ has been refractory to an initial shock. Two attempts at peripheral IV have been unsuccessful. The next recommended access route of administration for the delivery of drugs during CPR ^a^ is:**
a. External jugular vein
b. Femoral vein
c. Intraosseous
d. Endotracheal
**8. A patient with an ST-segment elevation MI ** ^**a**^ ** has ongoing chest discomfort. Fibrinolytic therapy has been ordered. Heparin 4000 U IV bolus was administered and a heparin infusion 100 U per hour is being administered, and Aspirin was not taken by the patient because he had a history of gastritis treated 5 years ago. Your next action is to:**
a. Substitute clopidogrel 300 mg loading dose
b. Give aspirin 160 – 325 mg chewed, immediately
c. Give 75 mg enteric-coated aspirin only
d. Give 325 mg enteric-coated aspiring rectally
**9. A patient with possible ACS ^a^ and a bradycardia of 42/min has ongoing chest discomfort. What is the initial dose of atropine?**
a. Atropine 0.5 mg
b. Atropine 1.0 mg
c. Atropine 0.1 mg
d. Atropine 3 mg
**10. A patient is in cardiac arrest. VFib has been refractory to an initial shock. Of the following, which drug and dose should be administered first by IV/IO route?**
a. Atropine 1 mg
b. Epinephrine 1 mg
c. Vasopressin 20 U
d. Sodium bicarbonate 50 mEq
**11. A 35-yearold woman has palpitations, lightheadedness, and a stable tachycardia. The monitor shows a regular narrow-complex QRS at a rate of 180/min. Vagal maneuvers have not been effective in terminating the rhythm. An IV has been established. What drug should be administered IV?**
a. Epinephrine 2-10 ụg/kg per minute
b. Atropine 0.5 mg
c. Lidocaine 1 mg/kg
d. Adenosine 6 mg
**12. A patient with a possible ST-segment elevation MI has ongoing chest discomfort. Which of the following would be a contraindication for administration of nitrates?**
a. HR of 90/min
b. BP > 180 systolic
c. Use of phosphodiesterase inhibitor within 12 hours
d. Left ventricular infarct with bilateral rales
**13. A patient has sinus bradycardia with a rate of 36/min. Atropine has been administered to a total dose of 3 mg. TCP ^a^ has failed to capture. The patient is confused and BP is 100/60. Which of the following is now indicated?**
a. give additional 1mg Atropine
b. Give NS bolus 250 mL-500mL
c. Start dopamine 10-20 ụg/kg per minute
d. Start epinephrine 2-10 ụg/min
**14. A patient is in pulseless VTach ^a^ . Two shocks and one dose of epinephrine have been given. The next drug/dose to anticipate to administer is:**
a. Vasopressin 40U
b. Amiodarone 150 mg
c. Lidocaine 0.5 mg/kg
d. Epinephrine 3 mg
e. Amiodarone 300 mg
**15. A patient is in refractory VFib and has received multiple appropriate defibrillations, epinephrine 1 mg IV twice, and an initial dose of lidocaine IV. The patient is intubated. A second dose of lidocaine is now called for. The recommended second dose of lidocaine is:**
a. 0.5 - 0.75 mg/kg IV push
b. 2-3 mg/kg IV push
c. Give endotracheal dose 2-4 mg/kg
d. Start infusion 1 - 2 mg/min
e. 1 mg/kg IV push
**16. You arrive on-scene with the Code Team. High-quality CPR is in progress. An AED ^a^ has previously advised “no shock indicated”. A rhythm now finds asystole. The next action you would take is to:**
a. place a Combitube or Laryngeal Mask Airway (LMA)
b. Attempt intubation with minimal CPR interruption
c. Call for a pulse check
d. Place IV or IO access
**17. Which of the following is the most accurate statement regarding the administration of vasopressin during cardiac arrest?**
a. Vasopressin is indicated for VF and pulseless VT prior to the delivery of the first shock
b. Vasopressin can be administered twice during cardiac arrest
c. Vasopressin is recommended instead of epinephrine for the treatment of asystole
d. The correct dose of Vasopressin is 40 U administered IV or IO
**18. A patient is in cardiac arrest. High-quality chest compression is being given. The patient is intubated and an IV has been established. The rhythm is asystole. The first drug/dose to administer is:**
a. Atropine 0.5 mg IV or IO
b. Epinephrine 3 mg via ETT
c. Dopamine 2 to 20 ụg/kg per minute IV or IO
d. Atropine 1 mg IV or IO
e. Epinephrine 1 mg or Vasopressin 40 U IV or IO
**19. A 57-yearold woman has palpitations, chest discomfort and tachycardia. The monitor shows a regular wide-complex QRS at a rate of 180/min. She becomes diaphoretic and BP is 80/60. The next action is to:**
a. Obtain 12 lead ECG
b. Perform immediate synchronized cardioversion
c. Establish IV and give sedation for electrical cardioversion
d. Give amiodarone 300 mg IV push
**20. A patient is in refractory VFib. High quality CPR is in progress and shocks has been given. One dose of epinephrine was given after the second shock. An antiarrhythmic drug was given immediately after the third shock. What drug should the team leader request to be prepared for administration?**
a. Repeat the antiarrhythmic
b. Escalating dose epinephrine 3 mg
c. Second dose of epinephrine 1 mg
d. Sodium bicarbonate 50 mEq
**21. A bradycardia rhythm istreated when:**
a. HR is < 60 with or without symptoms
b. BP < 100 systolic without symptoms
c. The patient has an MI on the 12-lead ECG
d. CP or shortness of breath is present

^a^ Abbreviations: ACS, acute coronary syndrome; AED, automated external defibrillator; BP, blood pressure; CPR, cardiopulmonary resuscitation; CT, computed tomography; ER, emergency room; IO, intraosseous; IV, intravenous; MI, myocardial infarction; TCP, trans-cutaneous pacing; VFib, ventricular fibrillation; VTach, ventricular tachycardia.

## 4. Results

In the present study, 43 interns of group II and 41 interns of group I were studied. There was no significant difference in terms of sex, age, education, and the scores of National Comprehensive Basic Sciences Examination (NCBSE) and National Comprehensive Pre-internship Examination (NCPE) (P > 0.05). The present educational intervention could raise the knowledge of interns about the routes for drug administration in both groups. Although there was an increase in the number of correct answers in the lecture-based education group, there was no significant difference compared with the pre-educational exam results (P = 0.49). According to the analyses, there was a significant difference between these two educational methods in terms of interns’ knowledge growth (P = 0.022). In other words, electronic learning was more efficient than lecture-based education regarding routes for CPR drug administration (Table 1). 

Generalized estimating equations (GEE) test showed that both interventions could raise the level of knowledge of interns about CPR drug dosage forms (P < 0.001) and a statistically significant difference was observed between two groups (P = 0.039). In other words, electronic learning was a better method in increasing knowledge of CPR drug dosages (Table 1). Implementation of educational interventions helped interns have more correct answers about clinical judgment and appropriate drug administration. GEE test also revealed that the rate of increase in correct answers of both groups was significant (P < 0.001). This shows that both educational methods may improve the level of knowledge. No significant differenceswere observed (P = 0.11) between these two educational strategies in terms of increased knowledge of students who participated in this study in the field of clinical judgment and appropriate drug administration used in resuscitation (Table 1). According to the GEE test, in the lecture-based group, the rate of improvement in correct answers about alternative drugs in emergency situations was not significantly different from pre-intervention answers (P = 0.62), while the effectiveness of electronic learning on interns’ knowledge was significant (P = 0.005). Despite a marked increase in the number of correct answers in the group I, no statistically significant difference (P = 0.49) was observed between the two methods (Table 1). In assessment of effectiveness of the two methods, we found that before educational intervention, the average score of answering to all 21 questions was 7.5 ± 2.6 (lecture-based education: 6.97 ± 2.25 vs. electronic learning: 7.58 ± 2.5) and there was no significant difference between the two groups (P = 0.55). After education, however, the mean total correct answers reached to 11.0 ± 3.9 (lecture-based education: 10.44 ± 3.68 and electronic learning: 11.87 ± 3.66) that had a statistically significant increase (P < 0.001). Though, the average increase in correct answers between the two groups was not statistically significant (P = 0.49).

**Table 1.** The Effectiveness of Educational Methods on Knowledge of Cardiopulmonary Resuscitation Drugs in Groups I and II

**Level of Knowledge**	**Before Education ^a^**	**After Education ^a^**	**Β ^a^**	**Robust SE ^b^**	**P Value ^c^**
**Routes for Drug Administration**					
Electronic learning (Group I)	1.4 ± 1.7	2.5 ± 1.0	-0.7	0.14	< 0.001
Lecture-based education (Group II)	1.7 ± 1.2	1.9 ± 1.2	-0.089	0.13	0.49
**Clinical Judgment and Appropriate Drug Administration**					
Group I	3.6 ± 1.9	5.3 ± 2.0	-0.39	0.081	< 0.001
Group II	3.9 ± 1.5	4.7 ± 2.05	-0.46	0.096	< 0.001
**Drug Dosage Forms**					
Group I	2.4 ± 1.35	4.7 ± 1.6	-0.668	0.088	< 0.001
Group II	2.5 ± 1.3	4.1 ± 1.4	-0.51	0.081	< 0.001
**Alternative Drugs**					
Group I (frequency)	15 ± 36	25 ± 61.0	0.996	0.353	< 0.005
Group II (frequency)	21 ± 48	23 ± 53.5	0.186	0.37	0.62

^a^ The data are expressed as mean ± SD, B reports Regression coefficient.

^b^ Abbreviation: SE, standard error.

^c^ Based on generalized estimating equations regression.

## 5. Discussion

The findings of our study revealed that electronic learning, in comparison with lecture-based education, is not more efficient or effective in terms of improvement of interns’ knowledge regarding CPR. Delasobera et al. compared three educational methods of simulation, multimedia, and ordinary teaching in learning advanced skills of CPR. They suggested that learning through computer-based simulation method (multimedia) was more efficient and durable than the two other methods (26). Romero et al. stated that web-based electronic learning played a key role in interns’ knowledge of CPR (27). According to Ko et al. in computer-based model, students had better comprehension of ACLS than in traditional method and additionally, the students were more comfortable with it (28).

Moreover, Cook et al. indicated that web-based training might result in improvement of clinical skills, and airway management, administration of defibrillation and other CPR skills (29). On the other hand, Monsieurs et al. believed that although electronic learning is a useful method in medical education, improvement of practical skills requires an alternative method (30). Furthermore, the study of Perkins et al. as the only study with similar results to ours, revealed that teaching advanced CPR interventions through electronic learning methods cannot improve the quality of education (31).

Based on the literature, there are considerable controversies over efficiency of electronic learning in the improvement of the clinical skills, due to the existing distinction between education systems and structures. It should be mentioned that most of these studies were indicators of positive sides of electronic learning in the improvement of the clinical skills of physicians and nurses and consequently the improvement of the provided services of this sector. As mentioned in the results section, mean and standard deviation of interns’ scores (from 21 questions) before education was 7.5 ± 2.6 that reached to 10.44 ± 3.68 after lecture-based education and to 11.87 ± 3.66 after electronic learning. Accordingly, mean scores were low, which could be the indicator of the weakness of the educational system as a result of inefficiency of the educational structure and system, inappropriate teaching environment, and incompatibility of curriculums with society’s and students’ needs. Thus, the current educational system needs drastic and serious revision. Education of the instructions required for CPR, seems to be of low effectiveness regarding the subject of this research. Some factors contribute to the inappropriate knowledge levels of the medical students in the management of patients that need CPR including the educational programs, atmosphere, and high volume of the taught subjects within a short time period, vigorous night shifts, and subsequent exhaustion of the students. We believe that in order to evaluate different educational methods, further influential variables should be taken into consideration. As an example, the effectiveness of an experienced compared with an inexperienced lecturer in using educational tools might be significantly higher, which in turn could contribute to the different results obtained from our study. Since our department is newly established, the lack of long-term follow-up and the small sample size could be considered as the limitations of the present study.

Electronic learning is not of further effectiveness in the improvement of interns’ knowledge of advanced CPR drug pharmacology compared to lecture-based method. Regardless of the teaching method, students have poor knowledge of the required interventions. Therefore, particular attention should be paid to this concern and effective measures should be taken in order to improve the curriculums and educational courses.

## References

[1] Shumway JM (2004). Components of quality: competence, leadership, teamwork, continuing learning and service.. Med Teach..

[2] Soleimanpour H, Panahi JR, Mahmoodpoor A, Ghafouri RR (2011). Digital intubation training in residency program, as an alternative method in airway management.. Pak J Med Sci..

[3] Soleimanpour H, Sarahrudi K, Hadju S, Golzari SEJ (2012). How to Overcome Difficult-Bag-Mask-Ventilation: Recents Approaches.. Emergen Med ..

[4] Christenson J, Nafziger S, Compton S, Vijayaraghavan K, Slater B, Ledingham R (2007). The effect of time on CPR and automated external defibrillator skills in the Public Access Defibrillation Trial.. Resuscitation..

[5] Sokouti M, Montazeri V (2009). Acute nonpenetrating tracheobronchial injuries: what is important in the mortality?. J Cardiovasc Thorac Res..

[6] Soleimanpour H, Tabrizi JS, Farnam A, Nikakhtar M, Mokhtarpour M, Golzari SE (2013). Attitudes of emergency medicine physicians towards family presence during resuscitation.. Resuscitation..

[7] Tavakoli N, Bidari A, Shams Vahdati S (2012). Serum Cortisol Levels as a Predictor of Neurologic Survival inSuccessfully Resuscitated Victims of Cardiopulmonary Arrest.. J Cardiovasc Thorac Res..

[8] Mahmoodpoor A, Soleimanpour H, Nia KS, Panahi JR, Afhami M, Golzari SE (2013). Sensitivity of palm print, modified mallampati score and 3-3-2 rule in prediction of difficult intubation.. Int J Prev Med..

[9] Saghizadeh M, Rahmani A, Ahangharzadeh Rezaie S (2006). Investigation of nurse's knowledge and practice working in ccu wards of taleghani hospital of Urmia university of medical sciences regarding adult cpr.. J Nurs Midwifery Faculty..

[10] Borimnejad L, Nikbakht Nasrabadi A, Mohammadi H (2008). The effect of cardiopulmonary resuscitation workshop on nurses’ sustained learning.. Iran J Med Educ..

[11] Hunziker S, Tschan F, Semmer NK, Zobrist R, Spychiger M, Breuer M (2009). Hands-on time during cardiopulmonary resuscitation is affected by the process of teambuilding: a prospective randomised simulator-based trial.. BMC Emerg Med..

[12] Motavaf M, Safari S, Alavian SM (2013). Understanding of Molecular Pain Medicine: Genetic Basis of Variation in Pain Sensation and Analgesia Response.. Anesth Pain Med..

[13] Pouraghaei M, Moharamzadeh P, Soleimanpour H, Rahmani F, Safari S, Mahmoodpoor A (2014). Comparison effect of Fentanyl – Sufentanil and Alfentanil on Hemodynamic at Rapid Sequence Intubation in the emergency department.. Anesth Pain Med..

[14] Soleimanpour H, Ziapour B, Negargar S, Taghizadieh A, Shadvar K (2010). Ventricular tachycardia due to flumazenil administration.. Pak J Biol Sci..

[15] Aghamohammadi D, Eydi M, Hosseinzadeh H, Amiri Rahimi M, Ej Golzari S (2013). Assessment of Mini-dose Succinylcholine Effect on FacilitatingLaryngeal Mask Airway Insertion.. J Cardiovasc Thorac Res..

[16] Ghaffarzadeh A, Shams Vahdati S, Salmasi S (2012). Assessment of emergency medicine residents' cardiopulmonary resuscitation team in imam reza hospital.. J Cardiovasc Thorac Res..

[17] Golzari SE, Khan ZH, Ghabili K, Hosseinzadeh H, Soleimanpour H, Azarfarin R (2013). Contributions of Medieval Islamic physicians to the history of tracheostomy.. Anesth Analg..

[18] Golzari SEJ, Soleimanpour H, Mehryar H, Salarilak S, Mahmoodpoor A, Rahimi Panahi J (2013). Comparison of three methods in improving bag mask ventilation.. Int J Prev Med..

[19] Hanci V (2012). Tracheal Intubation Without Use of Muscle Relaxants: Comparison of Remifentanil and Alfentanil.. Anesth Pain Med..

[20] Huikuri HV, Castellanos A, Myerburg RJ (2001). Sudden death due to cardiac arrhythmias.. N Engl J Med..

[21] Seyedhejazi M, Sheikhzadeh D, Sharabiani BA, Oskoue SS (2010). Cardiac arrest in a case of mucopolysaccharidosis after tracheostomy.. J Cardiovasc Thorac Res..

[22] Soleimanpour H, Gholipouri C, Panahi JR, Afhami MR, Ghafouri RR, Golzari SE (2011). Role of anesthesiology curriculum in improving bag-mask ventilation and intubation success rates of emergency medicine residents: a prospective descriptive study.. BMC Emerg Med..

[23] Soleimanpour H, Khoshnudi F, Movaghar MH, Ziapour B (2010). Improvement of decerebrate status in a hanged child following emergent tracheostomy.. Pak J Biol Sci..

[24] Soleimanpour H, Shams Vahdati S, Mahmoodpoor A, Rahimi Panahi J, Afhami MR, Pouraghaei M (2012). Modified cricothyroidotomy in skill laboratory.. J Cardiovasc Thorac Res..

[25] American Heart Association . (2006.). ACLS Pre-Course Self Assessment ..

[26] Delasobera BE, Goodwin TL, Strehlow M, Gilbert G, D’Souza P, Alok A, Raje P, Mahadevan SV (2010). Evaluating the efficacy of simulators and multimedia for refreshing ACLS skills in India. Resuscitation..

[27] Romero C, Ventura S, Gibaja EL, Hervas C, Romero F (2006). Web-based adaptive training simulator system for cardiac life support.. Artif Intell Med..

[28] Ko P, Scott J, Mihai A, Barus CE, Grant WD (2009). Comparison of simulation-based ACLS versus traditional ACLS curriculum in third-year medical students.. Simul Healthc..

[29] Cook NF, McAloon T, O'Neill P, Beggs R (2012). Impact of a web based interactive simulation game (PULSE) on nursing students' experience and performance in life support training--a pilot study.. Nurse Educ Today..

[30] Monsieurs KG, Vogels C, Bossaert LL, Meert P, Manganas A, Tsiknakis M (2004). Learning effect of a novel interactive basic life support CD: the JUST system.. Resuscitation..

[31] Perkins GD, Fullerton JN, Davis-Gomez N, Davies RP, Baldock C, Stevens H (2010). The effect of pre-course e-learning prior to advanced life support training: a randomised controlled trial.. Resuscitation..

